# Determination of strain pattern in patients with cardiac amyloidosis secondary to Multiple myeloma: a feature tracking study

**DOI:** 10.1186/1532-429X-18-S1-P273

**Published:** 2016-01-27

**Authors:** Sabha Bhatti, Srikanth Vallurupalli, Stephanie Ambach, Adam Z Magier, Abdul Hakeem, Wojciech Mazur

**Affiliations:** 1 grid.241054.60000000122929177Cardiology, UAMS, Little Rock, AR USA; 2 grid.414288.30000000404470683christ hospital, Cincinnati, OH USA

## Background

Cardiac MRI is frequently used in the diagnosis of cardiac amyloidosis. Feature tracking is a novel method of analyzing myocardial strain at the myocardial borders. We investigated myocardial deformation mechanics of both the right and left ventricles in patients with Multiple myeloma with suspected cardiac amyloidosis.

## Methods

Comprehensive strain analysis was performed in 46 patients with multiple myeloma with suspected cardiac amyloidosis. MRI strain by feature tracking was measured using 2D cardiac performance analysis MR software (Tomtec, Germany). Global longitudinal strain (GLS) was obtained obtained from averaging longitudinal strains of apical 4-, 2- and 3-chamber views. Global circumferential strain (GCS) was obtained from averaging circumferential strains of the basal, mid and apical short axis views. Endocardial and epicardial contours were analyzed separately. Right ventricular GLS was derived from apical 4 chamber view, only endocardial contour was analyzed.

## Results

Table 1 depicts the MR characteristics of the cohort. Normal LV wall thickness was present in 47% with biopsy proven cardiac amyloidosis. GLS and strain rate were significantly reduced in the epicardial and endocardial layers. There was no statistically difference in radial and circumferential strain as well as right ventricular GL strain. Segmental GL strain analysis showed typical apical sparing pattern in the endocardium but not in the epicardium(base to apex gradient: -6.2 ± 2 vs. -1.2 ± 0.2; p < 0.001).

## Conclusions

This feature tracking MRI analysis sheds light on strain mechanics in a cohort of early cardiac amyloidosis with a significant number of cases with normal LV wall thickness. Global longitudinal strain was affected earlier than radial and circumferential strain. Based on the pattern of apical sparing, amyloidosis affects epicardial strain earlier and without segmental variation compared to endocardial strain.

**Figure 1:**
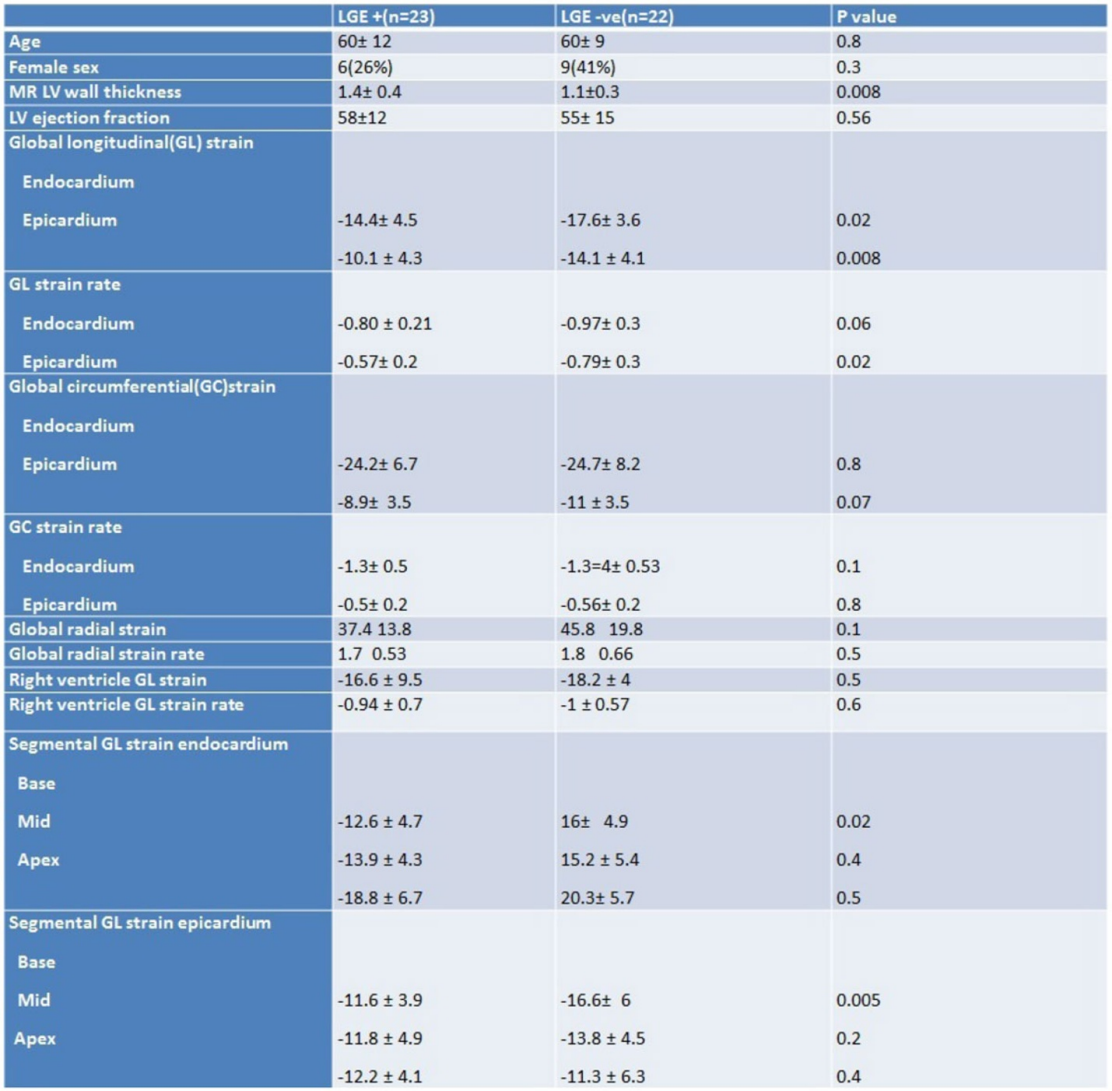
Figure 1

